# Antiviral Candidates and Vaccine Development for the Neglected Oropouche Virus

**DOI:** 10.3390/v18070754

**Published:** 2026-07-08

**Authors:** Vinicius Cardoso Soares, Suelen Silva Gomes Dias

**Affiliations:** 1 Laboratory of Immunopharmacology, Oswaldo Cruz Institute (IOC), Oswaldo Cruz Foundation (Fiocruz), Rio de Janeiro 21040-361, Brazil; 2 Center for Research, Innovation and Surveillance in COVID-19 and Health Emergencies, Oswaldo Cruz Foundation (Fiocruz), Rio de Janeiro 21040-361, Brazil; 3 Program of Immunology and Inflammation, Federal University of Rio de Janeiro UFRJ, Rio de Janeiro 21941-902, Brazil

**Keywords:** Oropouche virus, antiviral, *orthobunyavirus*, arbovirus, drug screening, natural products screening, repurposing drugs

## Abstract

The Oropouche virus (OROV), an orthobunyavirus primarily transmitted by the biting midge *Culicoides paraensis*, is the causative agent of Oropouche fever, a re-emerging arboviral disease associated with significant morbidity in Central and South America. The increasing frequency of outbreaks, including cases of sustained transmission in non-endemic regions and reports of vertical transmission, highlights the growing public health concern posed by OROV. Currently, there are no specific antiviral therapies or licensed vaccines available, underscoring the urgent need for effective therapeutic and preventive strategies. Recent advances in antiviral research have identified promising candidates, including repurposed drugs and bioactive compounds that target key stages of the viral replication cycle. In parallel, vaccine development has progressed through modern platforms, including viral vector-based and nucleic-acid-based technologies, enabling rapid responses to emerging outbreaks. However, major challenges remain, particularly due to the limited understanding of OROV pathogenesis, virus–host interactions, and the correlates of protective immunity. Furthermore, the ongoing evolution of OROV, including the genetic diversity and potential genomic rearrangements observed among circulating strains, represents an additional challenge that may influence viral characteristics and potentially affect the long-term efficacy of antiviral interventions and vaccine-induced protection. This review summarizes recent advances in the discovery of antiviral candidates and the development of vaccine approaches against OROV, both of which are essential for reducing the impact of OROV infections and strengthening preparedness for future outbreaks.

## 1. Introduction

Oropouche virus (OROV) is an enveloped, tripartite RNA virus belonging to the *Orthobunyavirus* genus within the *Peribunyaviridae* family and has recently emerged as a significant public health threat across the Americas. The virus is primarily transmitted to humans through the bite of the midge *Culicoides paraensis*, although certain mosquito species have also been implicated in its complex transmission cycles [1,2]. Clinically, Oropouche fever manifests as an acute febrile illness characterized by the sudden onset of high fever, severe headache, arthralgia, and myalgia, frequently leading to misdiagnosis with other co-circulating arboviral diseases such as dengue, zika, and chikungunya [3]. Rare complications include severe neurological manifestations, such as meningitis and encephalitis, with a recent increase in reported neuroinvasive cases, as well as an association with Guillain–Barré syndrome, reinforcing the need for enhanced surveillance and a better understanding of contemporary OROV strains [4,5].

Historically considered a neglected tropical pathogen confined to the Amazon Basin, OROV has been associated with an estimated 336,000 infections during periods of continuous endemic transmission and approximately 9.4 million infections during major outbreaks between 1960 and 2025 [6]. However, a significant expansion in the geographic distribution of OROV occurred between 2024 and 2025, with cases reported across multiple countries in Latin America and the Caribbean [7,8,9,10,11]. The actual burden of Oropouche fever is likely underestimated because of underreporting, particularly in these regions. In addition, travel-related cases have been documented in North America and Europe [12,13].

This recent epidemiological shift is underscored by alarming reports of the first documented OROV-related fatalities in two previously healthy young adults from a non-endemic region of Brazil outside the Amazon Basin, followed by two additional confirmed deaths, according to the 2024 epidemiological update for the Region of the Americas. Furthermore, as of August 2025, five deaths had been reported in Brazilian states, with two additional cases still under investigation, while one death was reported in Darién Province, Panama [14,15,16].

In July 2024, the Pan American Health Organization issued an Epidemiological Alert regarding the association between OROV infection and vertical transmission. OROV has been detected in multiple fetal compartments, including blood, cerebrospinal fluid, placenta, and umbilical cord tissue, as well as in internal organs such as the brain, heart, lungs, liver, kidneys, and spleen. Perinatal autopsy findings have provided evidence of significant central nervous system involvement in infants with congenital OROV infection, revealing severe abnormalities including microcephaly, ventriculomegaly, neuronal necrosis, and agenesis of the corpus callosum. According to recent reports from Brazil, there are currently three confirmed cases of vertical transmission, resulting in two fetal deaths and one congenital malformation, while investigations remain ongoing regarding OROV’s potential involvement in an additional 15 fetal deaths, three congenital malformations, and five spontaneous miscarriages [17,18,19]. Currently, the primary preventive measures against Oropouche fever remain vector control and personal protective strategies, including the use of insect repellents and window screens. However, the emergence of resistance in *Culicoides paraensis* populations to commonly used pyrethroid insecticides represents a substantial challenge to these traditional control strategies [1,20].

Despite the increasing clinical severity and epidemic potential of OROV, there are currently no approved antiviral therapies or vaccines, and patient management remains limited to supportive care [21]. Nevertheless, recent research has identified promising antiviral candidates currently under evaluation [22], while advances in vaccine development have generated encouraging results [1,23]. Due to its tripartite negative-sense RNA genome, OROV exhibits considerable genetic plasticity, facilitating evolution through mechanisms such as genetic reassortment, a hallmark of segmented orthobunyaviruses. Reassortment occurs when two distinct viral strains co-infect the same host-cell, enabling the exchange of genome segments and potentially generating novel lineages with altered biological properties [24,25]. The unprecedented 2023–2024 outbreak in the Americas has been associated with the emergence of a novel reassortant lineage containing an M segment derived from eastern Amazonian isolates and L and S segments originating from viruses previously circulating in Peru, Colombia, and Ecuador [26]. In addition to reassortment, OROV undergoes antigenic drift through the gradual accumulation of point mutations, further enhancing its capacity to adapt to new hosts and changing environmental conditions [27,28].

These genetic changes may have important phenotypic consequences, affecting viral replication efficiency, host immune responses, and pathogenic potential, while also posing significant challenges for the development of effective antiviral therapies and vaccines [24,25]. Contemporary outbreak isolates, including OROV-240023 and AM0088, exhibit enhanced replication kinetics in both human cell lines and insect vectors when compared with the historical Lineage I prototype strain BeAn19991. This increased fitness may be associated with sequence variations in the RNA-dependent RNA polymerase (RdRp), as well as polymorphisms within untranslated regions capable of modulating viral transcription, replication efficiency, and polymerase activity. These evolutionary adaptations are particularly concerning because they coincide with an expanded clinical spectrum observed during recent outbreaks, including the first reported human fatalities, severe neurological manifestations, and adverse pregnancy outcomes associated with vertical transmission [29,30,31].

Another major challenge is the historical lack of robust animal models capable of accurately reproducing the complexity of human OROV infection and disease progression. Consequently, in vitro experimental systems remain essential for studies of viral propagation, replication, and antiviral evaluation. Mammalian cell lines, including Vero cells (derived from African green monkey kidney epithelial cells) and Huh7 cells (derived from human hepatocellular carcinoma), are widely used because of their high permissiveness to OROV infection. Notably, Huh7 cells exhibit greater viral replication efficiency and faster growth kinetics than Vero cells, highlighting their value as a complementary in vitro model for investigating OROV biology and evaluating antiviral candidates, particularly given their relevance to OROV hepatotropism [32,33]. Concerning in vivo models, neonatal mice and immunocompromised strains, such as IFNAR−/− and IRF3/7/5−/− mice, are commonly used to investigate neuroinvasion and lethal disease because adult immunocompetent mice are naturally resistant to severe manifestations [33,34]. Syrian hamsters provide a naturally susceptible alternative that recapitulates systemic dissemination and overt clinical symptoms, including hind-limb paralysis [35], whereas non-human primates, including rhesus and pigtail macaques, support high-titer viremia and robust immune responses but generally remain asymptomatic [36].

However, each of these models presents important limitations. Immortalized cell lines may lack physiological relevance because of long-term adaptation to in vitro growth conditions, whereas immunocompromised mice fail to fully reproduce an intact human immune response. Furthermore, the availability of immunological reagents for hamster models remains limited, and the absence of clinical disease in non-human primates, combined with high costs and ethical constraints, reduces their usefulness as pathogenicity models. Importantly, most studies have focused on the historical BeAn19991 prototype strain, which may be laboratory-adapted and therefore display phenotypic characteristics distinct from those of recently emerged epidemic reassortant strains [22,32].

Therefore, this review aims to provide an updated overview of potential therapeutic strategies against OROV, focusing on the identification of promising candidates through drug-repurposing approaches and the evaluation of antiviral compounds targeting critical stages of the viral replication cycle. In parallel, advances in preventive strategies, including vaccine development, are also discussed. Ultimately, the successful control of OROV will depend on coordinated efforts to strengthen surveillance systems and expand research into antiviral and vaccine development.

## 2. Structure and Replication Cycle of OROV

OROV is a segmented negative-sense RNA virus belonging to the genus *Orthobunyavirus* and the family *Peribunyaviridae*. This enveloped virus, with a diameter ranging from 80 to 120 nm, possesses a tripartite genome consisting of three single-stranded RNA segments: Large (L), Medium (M), and Small (S). The L segment is approximately 6852 nucleotides long and encodes the RdRp, while the M segment is approximately 4385 nucleotides long and encodes the envelope glycoproteins Gn and Gc, as well as the non-structural protein of the M segment (NSm), which is thought to contribute to viral assembly and budding. Furthermore, the S segment is approximately 946 nucleotides in length and encodes the nucleocapsid (N) protein and the non-structural protein of the S segment (NSs), a key factor involved in immune evasion and viral pathogenesis [1,37,38].

The OROV replication cycle depends on endocytic entry and membrane fusion within acidified intracellular compartments. It begins with the binding of the viral surface Gn and Gc to specific host-cell receptors, including the low-density lipoprotein receptor-related protein 1 (LRP1) [39]. This interaction triggers clathrin-mediated endocytosis and subsequent formation of early endosomes. As the endosomal environment acidifies, the viral envelope fuses with the endosomal membrane, a process mediated by Gc, which functions as a class II viral fusion protein. Consequently, the viral ribonucleoprotein complexes (vRNPs) are released into the host-cell cytoplasm [40].

Intracellularly, RdRp initiates the primary transcription of the negative-sense genomic RNA (gRNA) to generate subgenomic mRNAs (sgRNA), which are subsequently translated by the host-cell machinery into viral proteins. RdRp employs a “cap-snatching” mechanism, whereby capped fragments derived from host mRNAs are used to prime viral mRNA synthesis [41]. Simultaneously, RdRp synthesizes full-length complementary RNA (cRNA) intermediates from the genomic RNA segments, which subsequently serve as templates to produce progeny genomic RNA [20].

**Figure 1:**
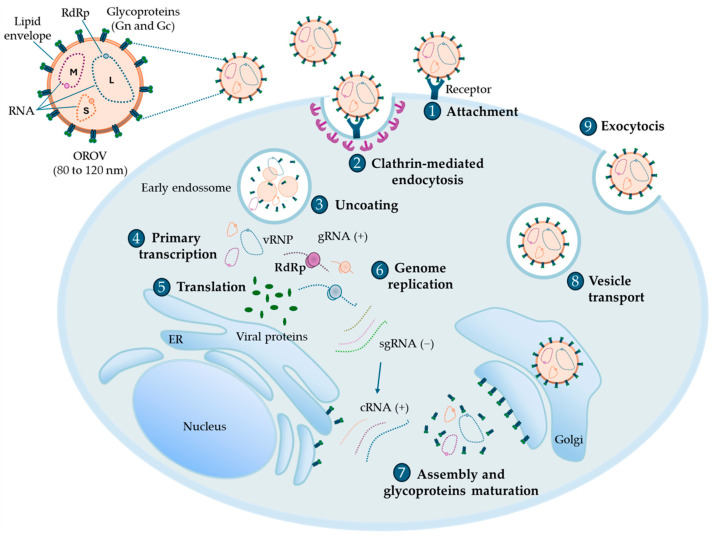
OROV replication cycle. (1) OROV attaches to specific receptors on the host-cell surface through its glycoproteins Gn and Gc, promoting viral entry. (2) The virus enters the host-cell through clathrin-mediated endocytosis. (3) The viral envelope subsequently fuses with the endosomal membrane, resulting in viral uncoating and the release of viral ribonucleoprotein complexes (vRNPs) into the host-cell cytoplasm. (4) The RNA-dependent RNA polymerase (RdRp) initiates the primary transcription of the negative-sense genomic RNA (gRNA), generating subgenomic viral mRNAs. (5) These viral mRNAs are subsequently translated by the host-cell machinery into viral proteins. (6) During viral mRNA synthesis, RdRp employs a “cap-snatching” mechanism, hijacking capped host mRNA fragments to prime viral transcription. The negative-sense genomic RNA is subsequently replicated through a positive-sense antigenomic RNA intermediate, which serves as a template for the synthesis of new genomic RNA segments. (7) Newly synthesized genomic RNAs and structural proteins are transported to the Golgi apparatus, where virion assembly occurs. (8) Newly formed viral particles are transported from the Golgi apparatus in secretory vesicles toward the plasma membrane. (9) Finally, mature virions are released from the host-cell via calcium-dependent exocytosis, enabling OROV dissemination and the infection of neighboring cells.

The final stages of the replication cycle involve the maturation of Gn and Gc within the endoplasmic reticulum–Golgi intermediate compartment and the Golgi apparatus. Newly synthesized genomic RNA associates with the N protein to form new vRNP complexes, which are then assembled into mature virions through Golgi-dependent budding. These mature particles are transported through the secretory vesicular trafficking pathway to the plasma membrane and released into the extracellular environment via calcium-dependent exocytosis (Figure 1) [20].

## 3. Identification of Direct-Acting Antivirals Against the RdRp of OROV

The development of antivirals that affect viral replication, particularly those targeting RdRp, has been extensively explored given their indispensable importance in the viral cycle [42]. In this context, direct-acting antivirals targeting the OROV RdRp have been identified and evaluated in preclinical models (Table 1).

### 3.1. Favipiravir

Favipiravir, (T-705), a purine or guanosine nucleoside analog, has been approved in Japan for the treatment of Severe Fever with Thrombocytopenia Syndrome (SFTS) caused by Dabie bandavirus, and it is already an approved drug for influenza treatment [45,48].

The primary mechanism through which favipiravir exerts its antiviral effect involves the direct inhibition of the OROV RdRp. By functioning as a chain terminator during the viral replication, the drug effectively halts the synthesis of the viral genome, preventing the formation of new infectious progeny [22]. Recent experimental efforts have utilized advanced reporter virus systems, such as recombinant OROV expressing enhanced green fluorescent protein (rOROV/GFP), to visualize and quantify this inhibitory effect in real-time in Huh7 cells. In these assays, favipiravir demonstrated a dose-dependent reduction in OROV replication, with an IC_50_ of 90.9 μM [32].

In a more recent study, favipiravir treatment demonstrated a stronger antiviral effect, with an EC_50_ of 28.4 μM in Vero cells. The drug exhibited a dose-dependent inhibition of OROV replication, leading to a reduction in the viral load, preservation of cellular morphology, and a progressive reduction in cytopathic effect (CPE) as drug concentrations increased [43].

Interestingly, oral administration of favipiravir initiated 1 h before infection provided complete protection against OROV infection in a Syrian hamster model, effectively limiting viral dissemination to peripheral organs and the central nervous system. It is important to note, however, that the in vitro antiviral activity reported in the same study was evaluated using a rOROV/GFP reporter system. Although this approach enables real-time monitoring of viral replication and facilitates antiviral screening, insertion of the reporter gene may alter viral replication kinetics or replication efficiency compared with wild-type OROV, representing a potential limitation when extrapolating these findings. Notably, its efficacy was maintained even when treatment was initiated after 24 h post-infection (hpi). Animals receiving a high dose of favipiravir (600 mg/kg/day) survived throughout the 26-day study period without exhibiting clinical signs of disease and showed consistent weight gain. In contrast, treatment with a lower dose of favipiravir (100 mg/kg/day) provided only partial protection [43].

The broad-spectrum potential of favipiravir is particularly relevant given the current absence of approved therapies for OROV, for which patient management remains limited to supportive care. By evaluating favipiravir alongside other candidates, researchers aim to determine its potential to suppress viral replication and establish a foundation for therapeutic strategies against OROV [32].

### 3.2. 4′-Fluorouridine

While favipiravir remains a promising candidate, comparative cell culture studies indicate that its inhibitory potency may be lower than that of emerging agents such as 4′-fluorouridine (4′-FlU; also known as EIDD-2749), a uridine analog containing a fluorine substitution at the 4′ position. Its active triphosphate metabolite (4′-FlU-TP) acts as an immediate chain terminator of the RdRp of influenza A virus while inducing transcriptional stalling in the RdRp of respiratory syncytial virus and Severe Acute Respiratory Syndrome Coronavirus 2 (SARS-CoV-2) [49,50].

Against OROV infection, the compound exhibited potent antiviral activity in the low-nanomolar range, with EC_90_ values ranging from 5.6 to 7.4 nM in Vero cells. The therapeutic potential of 4′-FlU is further underscored by its performance in IFNAR−/− mice, an interferon-α/β receptor knockout model, which serves as a highly susceptible preclinical model of lethal OROV infection [22,45]. Oral administration of 4′-FlU (0.3 to 10 mg/kg) for 7 consecutive days achieved near-complete survival when treatment was initiated 2 h before infection. Notably, the drug provided significant protection even when treatment with 3 mg/kg was initiated 72 hpi, and reduced viral titers in multiple compartments, including the serum, liver, and brain, representing an advanced stage of systemic infection. A critical feature of 4′-FlU is its apparent ability to cross the blood–brain barrier or effectively limit viral dissemination to the central nervous system. Unlike many other antiviral candidates, it successfully prevented the development of fatal encephalitis, reduced neuroinflammation, and protected brain tissue from OROV-induced damage [45].

Another study evaluated the antiviral activity of 4′-FlU against infection with an epidemic OROV strain in vitro. Treatment exhibited potent antiviral activity, inhibiting CPE with EC_50_ values of 0.012 μM in VeroE6 cells and 0.048 μM in JEG-3 cells while reducing viral RNA levels with EC_90_ values of 0.17 μM in A549 cells and 0.24 μM in JEG-3 cells. No detectable cytotoxicity was observed at concentrations up to 50 μM. Importantly, 4′-FlU demonstrated greater antiviral efficacy than other RdRp-targeting antivirals evaluated in the same study, including favipiravir, ribavirin and molnupiravir [44].

In an OROV infection model using AG129 mice (129/Sv mice with knockouts in both IFNα/β and IFNγ receptors), oral treatment with 4′-FlU (10 mg/kg, once daily), initiated at 24, 48, or 72 hpi and continued until 7 days post-infection (dpi), resulted in 100% survival and complete absence of clinical disease. Treatment initiated at 24 or 48 hpi reduced viral RNA levels in serum and tissues to undetectable levels or below the lower limit of quantification (LLOQ), whereas treatment initiation at 72 hpi still reduced viral RNA levels by 1 log_10_ in the brain and up to 7 log_10_ in the liver. Furthermore, no infectious virus was detected, and lung and liver architecture remained preserved [44].

Despite these highly encouraging preclinical findings, several important limitations remain. To date, no clinical data has evaluated the efficacy or safety of 4′-FlU, and future investigations are necessary in patients with OROV infection. In addition, the potential for long-term antiviral resistance has not been comprehensively assessed, particularly under prolonged drug exposure or selective pressure. Furthermore, the safety profile of 4′-FlU has not yet been established in vulnerable populations, including pregnant individuals and immunocompromised patients, which is especially relevant given the increasing evidence linking OROV infection to vertical transmission and severe disease in high-risk groups.

Considering these in vitro and in vivo findings, 4′-FIU appears to be a highly promising therapeutic candidate for the treatment of OROV infection. Further studies are warranted to evaluate its clinical potential, particularly as a prophylactic intervention for individuals at high risk of severe disease or during the early stages of infection.

### 3.3. Ribavirin

Ribavirin (RBV), a broad-spectrum nucleoside analog (1-D-ribofuranosyl-1,2,4-triazol-3-carboxamide), has demonstrated antiviral effect against multiple RNA viruses in vitro, including SARS-CoV-2 and hepatitis C virus (HCV) [51,52,53]. Initial studies evaluating RBV against OROV infection reported no significant protective effects in either Vero-cell cultures or murine models [54]. However, subsequent investigations yielded contrasting results, highlighting RBV as a potentially promising antiviral candidate in both in vitro and in vivo models of OROV infection [32,43].

Using advanced recombinant virus systems, such as rOROV/GFP, researchers demonstrated that RBV inhibits OROV infection in a dose-dependent manner, with a half-maximal IC_50_ of 10.5 μM in Huh7 cells compared with 90.9 μM for favipiravir. This antiviral effect occurred without significant host-cell cytotoxicity, suggesting that RBV specifically interferes with viral replication rather than inducing nonspecific cellular stress [32].

However, other studies report more modest antiviral activity, with EC_90_ values of approximately 60 μM in Huh7 cells [45] and EC_50_ values as high as 115.7 μM in Vero cells [43], indicating that antiviral potency may vary depending on the experimental model employed. In Syrian hamsters, RBV treatment at doses of 40 or 100 mg/kg/day provided partial protection and reduced viral loads in serum, liver, and spleen. Nevertheless, infectious viruses remain detectable in the brain at 6 and 8 dpi, suggesting incomplete viral clearance from the central nervous system and potentially explaining the neurological manifestations observed following cessation treatment [43]. Taken together, these findings indicate that the antiviral efficacy of RBV is highly dependent on the experimental model and generally requires relatively high inhibitory concentrations. Moreover, its inability to achieve complete viral clearance, particularly from the central nervous system, highlights important limitations that warrant further investigation before clinical translation can be considered.

### 3.4. Remdesivir

Remdesivir is a nucleotide analog prodrug approved for the treatment of several viral infections, including SARS-CoV-2. Following intracellular conversion to its active triphosphate metabolite (RTP), it is recognized by the viral RdRp and incorporated into nascent viral RNA as remdesivir monophosphate (RMP). After incorporation, the RdRp adds several additional nucleotides before stalling, ultimately disrupting RNA elongation through a delayed chain-termination mechanism [52].

The use of remdesivir against OROV remains relatively underexplored. Available evidence indicates a significant antiviral effect, although it remains unclear whether its mechanism of action is identical to that described for other RNA viruses. Notably, remdesivir exhibited superior antiviral activity, with an IC_50_ of 0.31 μM in Huh7 cells compared to 10.5 μM for RBV and 90.9 μM for favipiravir. Furthermore, remdesivir reduced the infectivity of different OROV strains (PE-IAM4637 and BeAn19991) in a dose-dependent manner, with IC_50_ values of 0.21 and 0.17 μM, respectively. These findings highlight the potential of remdesivir as an effective therapeutic candidate for the treatment of Oropouche fever [32]. Nevertheless, important limitations remain. The proposed antiviral mechanism against OROV has not yet been experimentally confirmed, and the current evidence is restricted to in vitro studies. Consequently, validation in appropriate in vivo models, together with assessments of long-term antiviral efficacy and the potential emergence of drug resistance, will be essential to establish the therapeutic value of remdesivir for OROV infection.

### 3.5. Monulpiravir

Molnupiravir exhibits a distinct mechanism of action in targeting the viral RdRp of OROV. The active metabolite of molnupiravir, β-D-N4-hydroxycytidine triphosphate (NHC), is incorporated by the viral RdRp in place of cytidine or uridine triphosphates. During subsequent rounds of replication, NHC promotes the misincorporation of guanine and adenine, leading to the accumulation of mutations in the viral RNA genome. As a result, molnupiravir increases the mutational burden, driving the virus toward error catastrophe (lethal mutagenesis) and thereby inhibiting viral replication [52,55].

A recent study demonstrated that human-liver-derived organoids are highly permissive to OROV infection, establishing them as a physiologically relevant in vitro model for investigating viral pathogenesis and evaluating antiviral candidates. This model enables researchers to assess antiviral efficacy while simultaneously evaluating potential cytotoxic effects. Using this system, the antiviral activities of molnupiravir and its active metabolite, NHC, were investigated. Both compounds exhibited robust dose-dependent activity against the OROV-2024 isolate (IC_50_ of 1 μM for molnupiravir and 0.05 μM for NHC) and the OROV-1967 strain (IC_50_ of 2.2 μM for molnupiravir and 0.05 μM for NHC). Treatment effectively inhibiting viral replication, reducing CPE, and restoring cellular homeostasis in infected organoids [46].

These findings were further corroborated in human-fetal-liver-derived organoids, which were also susceptible to OROV infection and exhibited strong response to NHC treatment. This observation is particularly relevant given the detection of OROV RNA in the liver tissue of a stillborn infant during the 2023–2024 outbreak [56]. Nevertheless, several important limitations should be considered. Although liver-derived organoids represent a more physiologically relevant model than conventional cell lines, they do not fully recapitulate the complexity of the human liver, particularly with respect to cellular diversity, tissue architecture, vascularization, and immune cell interactions. Furthermore, the antiviral efficacy of molnupiravir and NHC has not yet been validated in in vivo models of OROV infection, and the precise mechanisms underlying their antiviral activity against OROV remain to be experimentally confirmed. Addressing these limitations will be essential for determining the translational potential of these compounds for the treatment of OROV infection.

### 3.6. Acridones

Acridones are pharmacologically active compounds characterized by a planar tricyclic aromatic structure that facilitates interactions with viral components, particularly nucleic acids. Their antiviral activity against OROV appears to involve a dual mechanism targeting both viral RNA and essential viral proteins. Acridones can intercalate into double-stranded RNA (dsRNA), disrupting viral RNA structure and impairing replication and transcription processes [14,20,47]. Additionally, these compounds interfere with the cap-snatching mechanism, a critical step in bunyavirus transcription [20,41,57].

FAC21 demonstrated potent antiviral activity against OROV, inhibiting approximately 95% of viral endonuclease activity, with an IC_50_ of 1.4 μM in Vero cells. Biochemical and computational analyses revealed that FAC21 acts as a competitive inhibitor by binding to the active site of the viral endonuclease, thereby blocking host mRNA cap cleavage and preventing viral mRNA synthesis. In contrast, FAC22 exhibited dsRNA intercalation activity but lacked significant endonuclease inhibition, highlighting the structural specificity required for antiviral activity [47,57].

Acridones also markedly reduced OROV particle release, achieving up to 90% inhibition between 8 and 48 h post-treatment, indicating a substantial impact on viral replication and progeny virion production. The distinct inhibition kinetics observed for FAC21 and FAC22 suggest different interactions with viral or cellular targets, further supporting their potential as antiviral candidates [47]. Given their broad-spectrum antiviral activity against both RNA and DNA viruses, acridones represent promising compounds for further investigation against OROV and other medically relevant viral pathogens [58,59]. Although inhibition of nucleic acid synthesis appears to be a major antiviral mechanism, additional pathways contributing to viral suppression remain to be fully elucidated. Nevertheless, important limitations remain before these compounds can be considered for clinical development. To date, their antiviral efficacy has not been validated in in vivo models of OROV infection, and key pharmacokinetic properties, including absorption, distribution, metabolism, and elimination, have not yet been characterized. Furthermore, comprehensive safety and toxicity assessments in animal models are still lacking, underscoring the need for additional preclinical studies to establish their translational potential as anti-OROV therapeutics.

## 4. Natural Compounds with Antiviral Potential Against OROV

Furthermore, naturally derived compounds, particularly those isolated from medicinal plants, have attracted increasing attention because of their potential antiviral activity against OROV. These bioactive molecules have been investigated for their ability to interfere with different stages of the viral replication cycle, highlighting natural products as promising sources for the development of novel therapeutic strategies against OROV infection. In this context, the viral Gn and Gc represent attractive targets for antiviral intervention because of their essential roles in viral attachment and membrane fusion. Furthermore, the host protease-mediated cleavage of the glycoprotein precursor into Gn and Gc is a critical maturation step that could be targeted to prevent the formation of infectious virions. Another promising therapeutic strategy involves disrupting the interaction between viral RNA and the N protein, which is essential for the formation of the vRNP and subsequent viral packaging (Table 2).

### 4.1. Quercetin Hydrate

Quercetin hydrate, a natural flavonoid belonging to the flavonol subclass, is abundantly found in a variety of plant-derived foods and has long been recognized for its diverse biological activities, including potent antioxidant and anti-inflammatory properties [64]. In the context of viral infections, quercetin has demonstrated broad-spectrum antiviral activity against several medically relevant viruses, such as dengue virus, zika virus and Mayaro virus [65,66,67].

In experiments using Vero cells, the compound exhibited significant antiviral activity against OROV, with an EC_50_ of 53.5 μM when administered 1 h after infection and evaluated at 48 hpi. These findings suggest that quercetin hydrate can effectively interfere with the viral replication cycle even after viral entry has occurred. Furthermore, treatment exhibited low cytotoxicity in this cellular model, highlighting the potential of quercetin hydrate as a promising and apparently safe antiviral candidate under in vitro conditions [60].

To elucidate the molecular mechanisms underlying this antiviral activity, in silico approaches, including molecular docking and molecular dynamics simulations, were employed. These computational analyses identified the OROV Gc as a primary target for interaction with quercetin hydrate, making it an attractive target within the OROV replication cycle [60]. By binding to specific pockets within the Gc, quercetin hydrate may interfere the conformational changes required for membrane fusion and the subsequent release of the viral genome into the host-cell cytoplasm.

The results obtained with quercetin hydrate indicate promising antiviral activity against OROV, as demonstrated by both in silico and in vitro analyses, which also provided insights into its potential mechanism of action. Nevertheless, important limitations should be acknowledged. The proposed antiviral mechanism has not yet been experimentally confirmed, and the current evidence is limited to cell-based and computational models. Furthermore, the antiviral efficacy of quercetin hydrate has not been validated in in vivo models of OROV infection. Therefore, additional mechanistic studies, together with preclinical in vivo investigations and, ultimately, clinical studies, will be required to establish its therapeutic potential and clinical applicability.

### 4.2. Lysergol

Lysergol was identified through an extensive phenotypic high-throughput screening of 7.784 compounds conducted during the critical 2023–2024 outbreak in Brazil. This natural ergot alkaloid, traditionally derived from *Claviceps* species or the plant *Ipomoea hederacea*, exhibited remarkable antiviral potency. Reductions in viral titers ranging from 10,000- to 100,000-fold were observed at concentrations between 2.4 and 9.6 μM, with significant antiviral activity against OROV detected at concentrations as low as 0.6 μM in T24 cells [61].

Mechanistic studies using OROV minigenome systems reveal that lysergol acts specifically during the early intracellular stages of the viral replication cycle by inhibiting the function of both RdRp and N protein [61]. Notably, the compound demonstrates a dose-dependent reduction in viral replication, reaching approximately 40% inhibition at 4.8 μM and increasing to 70% at 9.8 μM in Huh7 cells, while maintaining a high genetic barrier to resistance even after eleven sequential passages. Furthermore, no significant cytotoxicity was observed at the tested concentrations in either cell line, indicating a favorable in vitro safety profile [61].

However, the translation of lysergol from a promising experimental compound to a clinically applicable antiviral will require substantial additional investigation. To date, its antiviral efficacy has not been validated in vivo models of OROV infection, and its pharmacokinetic profile, including absorption, distribution, metabolism, and elimination, remains unknown. Likewise, comprehensive safety and toxicity evaluations in animal models are still lacking. These limitations are particularly relevant because lysergol belongs to the ergot alkaloid class, whose members are well known for their vasoconstrictor and uterotonic properties. Consequently, its therapeutic use would require careful safety assessment, especially in vulnerable populations such as pregnant individuals, given the growing evidence linking OROV infection to vertical transmission and adverse fetal outcomes [61].

### 4.3. Wedelolactone

Wedelolactone (WDL) is a natural compound found in plants such as *Eclipta alba* and is known to inhibit viral proteins and interfere with multiple stages of replication cycles in HCV, cytomegalovirus and herpes simplex virus [68,69,70]. In silico analyses against OROV have suggested that the primary mechanism of WDL activity involves a potent interaction with viral RdRp. More specifically, WDL acts as a viral endonuclease inhibitor [62].

Recent investigations into the antiviral potential of WDL have demonstrated that this natural bioactive compound effectively inhibits OROV replication in vitro. Using Vero cells, WDL efficiently inhibited viral endonuclease activity, achieving 100% inhibition at a concentration of 25 μM and exhibiting an IC_50_ of 310 nM [62].

These findings highlight WDL as a promising, accessible, and potentially low-toxicity alternative to conventional synthetic antiviral agents [62,71]. Notably, treatment with WDL at 40 μM in Vero cells throughout the post-inoculation period resulted in a dose-dependent reduction in viral titers, with an EC_50_ of 18.92 μM. This inhibitory effect was sustained for up to 48 hpi, suggesting that WDL interferes with early stages of the viral replication cycle [62].

Despite these promising in vitro findings, several important limitations remain. To date, the antiviral efficacy of WDL has not been validated in in vivo models of OROV infection, and its pharmacokinetic properties, including absorption, distribution, metabolism, and elimination, have not yet been characterized. Furthermore, comprehensive safety and toxicity evaluations in animal models are still lacking. Addressing these limitations will be essential to determine the translational potential of WDL as a therapeutic candidate for OROV infection.

### 4.4. Acylphloroglucinols and Xanthohumol

The investigation of acylphloroglucinols and xanthohumol from hops (*Humulus lupulus L*.) is particularly relevant because these compounds are widely utilized in the brewing industry and have a well-established record of safety and biological activity, including anti-inflammatory and broad-spectrum antiviral properties [63,72].

Experimental studies have demonstrated that these compounds exert significant inhibitory effects against OROV in both in vitro and in silico models. A subset of acylphloroglucinols known as beta acids exhibited potent antiviral activity in post-treatment assays, with an EC_50_ of 26.7 μM in Vero cells. Similarly, xanthohumol demonstrated antiviral activity, with an EC_50_ of 50.2 µg/mL in Vero cells. Unlike compounds that exclusively target viral entry, these molecules appear to interfere at multiple stages of the viral cycle, including viral replication, assembly, and release. This multi-stage inhibitory activity suggests that hop-derived compounds may provide a more robust antiviral effect by suppressing the production of progeny virions within infected cells. Molecular dynamics simulation revealed that these compounds exhibit high binding affinity for the OROV endonuclease [63,72].

Despite these encouraging findings, several important limitations remain. The antiviral activity of acylphloroglucinols and xanthohumol has not yet been validated in in vivo models of OROV infection, and their pharmacokinetic properties have not been characterized in the context of antiviral therapy. Furthermore, the proposed molecular targets are based primarily on computational predictions and therefore require experimental confirmation through biochemical and virological studies. Addressing these limitations will be essential to establish the translational potential of these hop-derived compounds as therapeutic candidates against OROV.

**Figure 2:**
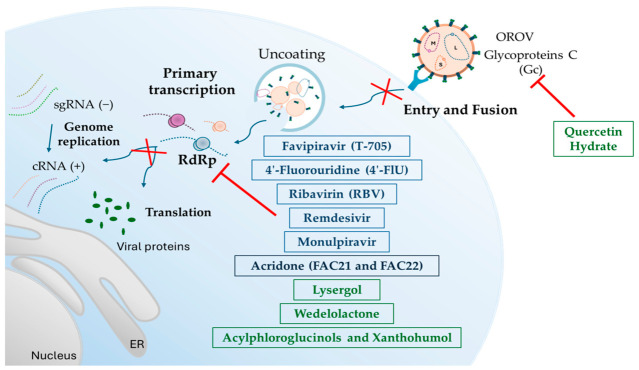
Schematic representation of the potential antivirals identified to date and their respective targets within the OROV replication cycle. Antivirals highlighted in blue correspond to synthetic compounds targeting the viral RNA-dependent RNA polymerase (RdRp), whereas those highlighted in green represent natural compounds targeting either RdRp or the OROV Gc.

Therefore, given the growing public health impact of OROV infection, understanding the molecular targets of antiviral therapies remains essential. In the absence of licensed vaccines and specific antiviral treatments, patient management remains largely supportive and focused on symptom relief. This therapeutic gap underscores the urgent need to develop targeted antiviral agents and repurpose existing compounds that effectively disrupt the OROV replication cycle and reduce disease severity (Figure 2).

Among the antiviral agents evaluated, 4′-FlU exhibited the highest potency against OROV, with EC_50_ values ranging from 0.012 to 0.048 μM across different cell lines and no detectable cytotoxicity at concentrations up to 50 μM. Remdesivir and molnupiravir were the next most potent compounds, exhibiting antiviral activity in the sub-micromolar-to-low-micromolar range. In contrast, ribavirin, favipiravir, and acridone derivatives required substantially higher concentrations to inhibit OROV replication (Table 3). Among the naturally derived compounds evaluated against OROV, WDL exhibited the highest antiviral potency, with an IC_50_ of 310 nM and a CC_50_ of 373.5 μM, indicating a favorable selectivity index. Lysergol also demonstrated promising antiviral activity at sub micromolar concentrations, although quantitative IC_50_ or EC_50_ values have not yet been established. In contrast, quercetin hydrate, acylphloroglucinols, and xanthohumol exhibited antiviral activity only at substantially higher concentrations. Nevertheless, the antiviral potency of wedelolactone remained lower than that of 4′-FlU, the most active synthetic antiviral identified to date (EC_50_ = 0.012 μM). Overall, 4′-FlU appears to be one of the most promising antiviral candidates for OROV, combining exceptional in vitro potency with robust in vivo efficacy and a favorable preclinical safety profile (Table 3).

## 5. Host-Directed Antiviral Strategies Against OROV: Immunomodulators

Viruses are obligate intracellular parasites that interact with multiple host-cell structures and manipulate key cellular signaling pathways to support their replication and persistence. In this context, host-directed therapies have emerged as a promising antiviral strategy, complementing the development of direct-acting antivirals by targeting host biological pathways that are essential for viral replication and pathogenesis. This approach is particularly attractive for orthobunyaviruses, whose high mutation rates may facilitate to the emergence of resistance to direct-acting antivirals.

### 5.1. Acetohexamide and Deptropine

Accordingly, a computational study identified acetohexamide and deptropine as potential host-directed therapeutic candidates against OROV. Using a system biology-based approach, the authors integrated network pharmacology and molecular docking analyses to identify approved drugs with repurposing potential OROV infection [73].

Detailed computational analyses revealed that both acetohexamide, a sulfonylurea traditionally used for the treatment of diabetes, and deptropine, an antihistamine, exhibit strong binding affinities for key host proteins implicated in OROV pathogenesis. Specifically, these compounds were predicted to interact with immunomodulatory proteins involved in inflammatory and antiviral responses, including Interleukin 10 (IL-10), Fas Ligand (FASLG), Protein Tyrosine Phosphatase Receptor Type C (PTPRC), and Fc Gamma Receptor IIIa (FCGR3A). By targeting these components within the host protein–protein interaction network, acetohexamide and deptropine may attenuate the excessive inflammatory response, often referred to as a cytokine storm, associated with severe Oropouche fever and its neurological complications [73,74,75].

The identification of these immune-related targets suggests that OROV may employ immune-modulatory strategies similar to those described for other arboviral infections [76]. This finding further highlights the therapeutic potential of host proteins involved in OROV pathogenesis. Although additional in vitro and in vivo studies are required to validate their efficacy and clinical relevance, these results reinforce the importance of advancing rational host-directed antiviral drug discovery strategies [22,73].

### 5.2. Type I Interferon (IFN-I)

Complementing these findings, early studies investigating innate immunity identified IFN-α as a critical antiviral mediator during OROV infection. IFN-α exhibited potent antiviral activity in the brains of OROV-infected mice and against other related orthobunyaviruses, particularly when administered prophylactically 24 h before infection, highlighting its potential as a host-directed antiviral strategy [77].

In another study, transcriptomic analyses performed using a human-liver-derived organoid model revealed robust virus–host interactions associated with the activation of interferon-stimulated genes (ISGs), including Interferon Alpha Inducible Protein 6 (IFI6), Interferon-Induced Protein with Tetratricopeptide Repeats 1 (IFIT1), Interferon-Stimulated Gene 15 (ISG15), and MX Dynamin-Like GTPase 1 (MX1). This model is particularly relevant because patients with OROV infection frequently exhibit elevated biomarkers of liver injury. Furthermore, pharmacological inhibition of the interferon signaling pathway enhanced OROV replication, whereas treatment with therapeutic IFN-α effectively suppressed viral infection [46]. Moreover, combination therapy with molnupiravir and IFN-α produced pronounced synergistic antiviral activity, underscoring the potential of combining virus-targeted and host-directed therapeutic strategies to achieve greater antiviral efficacy than either monotherapy alone [46].

Another important study demonstrated the essential role of the IFN-I response in maternal tissues during OROV infection. The authors showed that intact IFN-I signaling is crucial for controlling viral replication and limiting viral dissemination across the maternal–fetal interface. Conversely, impairment of the IFN-I pathway increased viral burden and facilitated viral spread between maternal and fetal tissues, suggesting a potential contribution to adverse pregnancy outcomes. These findings reinforce the importance of innate immune responses, particularly IFN-mediated antiviral mechanisms, in restricting OROV pathogenesis during pregnancy. Nevertheless, additional studies are required to identify the host and viral factors involved in the association between OROV infection and pregnancy-related complications [78].

Collectively, these findings demonstrate that OROV is highly sensitive to host innate immune signaling and suggest that therapeutic strategies aimed at enhancing type I interferon responses may represent an effective approach for limiting viral replication during the early stages of infection.

## 6. Potential Vaccines in Development for OROV and Associated Challenges

Developing safe, effective, and accessible vaccines against OROV represents a critical public health priority for addressing this neglected emerging threat. The absence of licensed vaccines continues to place a substantial burden on healthcare systems in affected countries. Accordingly, several innovative vaccine strategies have recently emerged to address this unmet public health need.

Pioneering studies established the first reverse genetics system for OROV, successfully generating recombinant viruses lacking either the NSm or NSs proteins. These viral proteins play important roles in OROV pathogenesis. NSm has been implicated in viral morphogenesis and budding, whereas NSs functions as a major virulence factor by antagonizing interferon-mediated antiviral responses and suppressing host-cell transcription, thereby promoting immune evasion [38,79]. Similar attenuation strategies have been successfully applied to other orthobunyaviruses, including Rift Valley fever virus, in which deletion of NSs and NSm generated genetically stable, attenuated, and immunogenic vaccine candidates capable of inducing protective immunity without causing clinical disease [80,81]. Therefore, the generation of OROV mutants lacking these virulence-associated factors provides a promising platform for the development of live-attenuated vaccines with improved safety profiles and the potential to induce robust and long-lasting immune responses.

In parallel with reverse genetics approaches, immunoinformatic-based strategies have emerged as valuable tools for rational vaccine design. Computational analyses have been employed to identify highly antigenic regions within the OROV polyprotein, including potential B-cell and T-cell epitopes with strong immunogenic potential. These approaches have also enabled the prediction of three-dimensional protein structures and the identification of potential ligand-binding sites through molecular docking analyses [82].

Such in silico approaches provide a rapid and cost-effective framework for antigen selection, particularly for neglected viral diseases, in which conventional vaccine development is often constrained by financial and technological limitations. However, despite these advantages, epitope-based vaccine candidates still require extensive experimental validation to confirm their immunogenicity, safety, and protective efficacy [82,83].

Among the most advanced preclinical vaccine platforms evaluated against OROV is a replication-competent chimeric vesicular stomatitis virus (VSV)-based vaccine expressing the OROV glycoprotein complex. The VSV-OROV chimera induced robust neutralizing antibody responses, including antibodies directed against the N-terminal region of the Gc. Importantly, vaccination significantly reduced viral replication and tissue viral loads in mice challenged with wild-type OROV, providing the first experimental evidence that targeting OROV surface glycoproteins may represent an effective vaccination strategy [84].

The VSV vector platform offers several advantages, including low levels of pre-existing vector immunity, the absence of genomic integration into host-cells, and a high capacity for the incorporation of foreign genetic sequences. Furthermore, this platform has already demonstrated clinical success through the rVSV-ZEBOV (Ervebo) vaccine against Ebola virus disease, which was approved by the European Medicine Agency in November 2019 and by the U.S. Food and Drug Administration in December 2019. This vaccine demonstrated very high efficacy in a Phase III ring vaccination trial conducted during the West Africa outbreak. Moreover, a single dose of the rVSV-ZEBOV (Ervebo) induces durable antibody responses for up to five years, supporting its utility for outbreak control despite gradual decline in antibody levels over time [85,86].

More recently, human codon-optimized messenger RNA–lipid nanoparticle (mRNA-LNP) vaccines encoding the OROV envelope glycoproteins from both a historical prototype lineage (BeAn19991, Brazil) and a recently circulating outbreak lineage (AM0059, Brazil) have emerged as highly promising vaccine candidates. Preclinical studies demonstrated that these vaccines provided complete protection against lethal OROV challenge in BALB/c and A129 mice by inducing robust humoral and cellular immune responses, including high titers of OROV-specific IgG antibodies and potent CD4^+^ and CD8^+^ T-cell responses. Notably, vaccines based on contemporary outbreak-associated sequences also protected against prototype viral strains, suggesting that incorporating updated antigenic sequences may enhance cross-lineage protection [87]. The flexibility of mRNA-LNP platforms, which enables the rapid modification in antigen sequences according to circulating viral variants, represents a major advantage for addressing the genetic diversity and evolutionary dynamics of OROV [23,87].

Despite these advances, several challenges remain for the successful development of an effective OROV vaccine. One important limitation is the lack of high-resolution structural information on the viral surface Gn and Gc, which restricts the rational identification of optimal antigenic targets and raises questions regarding whether future vaccines should target the glycoproteins, the nucleocapsid, or combinations of multiple viral antigens [23].

Genomic surveillance has identified amino acid substitutions in surface-exposed regions of the Gc among contemporary OROV strains, which may contribute to immune escape from antibodies elicited against ancestral lineages and underscore the need for lineage-matched vaccines or broadly neutralizing antibody-based approaches. Importantly, recent studies have identified antigenic differences between the historical prototype strain BeAn19991 and contemporary outbreak-associated lineages, such as AM0059. These findings suggest that vaccines based exclusively on ancestral strains may exhibit reduced neutralizing activity against currently circulating viruses, emphasizing the importance of incorporating updated antigenic sequences or developing broadly protective vaccine strategies [87].

Animal models remain essential for understanding OROV pathogenesis and evaluating vaccine efficacy. Although immunocompetent adult mice generally do not develop severe disease following infection, IFNAR−/− mice and Syrian hamsters are widely used as relevant models for investigating viral replication, disease mechanisms, and vaccine-induced protection [33,35]. Furthermore, non-human primates support efficient viral replication and mount immune responses that more closely resemble those observed in humans, providing an important translational model. An important research priority is determining whether maternal vaccination can prevent vertical transmission and fetal complications, particularly in light of recent evidence demonstrating OROV infection in human placental explants and trophoblast organoid models [22].

Additionally, vaccine candidates must demonstrate favorable safety profiles across diverse populations, including pregnant individuals, given previous concerns associated with vaccine development against other orthobunyaviruses [81]. Another critical consideration is the establishment of scalable, affordable, and sustainable manufacturing platforms to ensure vaccine accessibility in endemic regions with limited resources. Although several vaccine candidates have demonstrated promising immunogenicity and protective efficacy in animal models, none has yet advanced to clinical evaluation in humans [23].

The clinical diagnosis of OROV infection remains challenging because its symptoms frequently overlap with those of other co-circulating arboviral diseases in endemic regions, particularly dengue and yellow fever, making clinical diagnosis difficult [88]. Consequently, misdiagnosis may lead to inappropriate clinical management, delays in public health interventions, and underestimation of viral circulation.

Accurate and rapid identification of OROV infection is essential for mapping outbreak areas, monitoring viral dissemination, and implementing targeted control strategies, including vector-control measures and public health awareness campaigns. Recently, mouse monoclonal antibodies developed for OROV detection in indirect immunofluorescence assays and immunohistochemistry have shown considerable promise as diagnostic tools [89]. Although the two anti-OROV monoclonal antibodies exhibited promising reactivity patterns, they have not yet been evaluated against all four recognized OROV genotypes. Therefore, it remains unclear whether these antibodies can detect all currently circulating OROV lineages in South America. Nevertheless, the monoclonal antibody 63B3E7 recognizes a linear epitope within the nucleocapsid protein, one of the most conserved viral proteins among orthobunyaviruses, suggesting its potential broad applicability for OROV detection. Overall, these monoclonal antibodies represent valuable and versatile tools for OROV research, with applications in differential diagnosis, epidemiological surveillance, and studies of viral biology and pathogenesis [89].

The development of an effective OROV vaccine is essential to control future outbreaks and reduce the impact of this emerging arboviral disease. The combination of arthropod-borne transmission, increasing human mobility, and the expanding geographic distribution of OROV creates a significant risk of further dissemination beyond traditionally endemic regions [84]. A licensed vaccine would therefore represent a valuable preventive strategy, with the potential to reduce disease burden, prevent severe clinical manifestations, and protect vulnerable populations [74,90].

Vaccination strategies would be particularly relevant for communities in endemic regions of South and Central America, where recurrent outbreaks place considerable pressure on healthcare systems and contribute to substantial social and economic burdens [23,72]. In parallel with vaccine development, strengthening genomic surveillance, improving early diagnostic capacity, and enhancing epidemiological surveillance will remain essential components of integrated public health strategies aimed at controlling OROV transmission and limiting its future geographic spread. Collectively, these efforts will be critical for reducing the global impact of OROV infection and strengthening preparedness against future outbreaks of this re-emerging arbovirus.

## 7. Conclusions and Future Directions

In conclusion, OROV has emerged as an increasingly important public health concern in the Americas, driven by its expanding geographic distribution, the widespread distribution of competent vectors, and the large proportion of susceptible individuals. In this context, the development of effective antivirals and safe, effective vaccines is essential. Strategies such as drug repurpose and the identification of viral and host therapeutic targets offer substantial reductions in development time and cost, lower the risk of toxicity-related failures, and enable more rapid therapeutic responses, which are particularly important during outbreaks.

At the same time, important limitations must be acknowledged. Our current understanding of OROV biology, including its pathogenesis and the immune mechanisms underlying protective immunity, remains incomplete, directly limiting the rational design of both antiviral agents and vaccine candidates. Furthermore, the impact of viral genetic diversity on antiviral efficacy and vaccine performance has not yet been comprehensively evaluated.

OROV possesses a tripartite segmented RNA genome, which facilitates frequent genetic reassortment, as demonstrated by the 2023–2024 outbreak lineage containing an eastern Amazonian M segment combined with L and S segments derived from viruses circulating in Peru, Colombia, and Ecuador. This novel reassorting lineage exhibits enhanced replicative fitness and increased virulence compared with the historical Lineage I prototype strain BeAn19991. Such genetic variation represents a major challenge for therapeutic and vaccine development, as sequence divergence within the Gc may promote immune escape and reduce susceptibility to neutralizing antibodies elicited against ancestral strains.

Furthermore, although several antiviral candidates have shown promising activity, their mechanisms of action remain incompletely characterized in many cases. Among the compounds evaluated, 4′-fluorouridine (4′-FlU) stands out as one of the most promising antiviral candidates against OROV, combining sub nanomolar-to-low-nanomolar antiviral potency, high selectivity, and complete protection in animal models. In contrast, remdesivir and molnupiravir exhibit potent in vitro antiviral activity but still require validation in in vivo models of OROV infection. Although favipiravir and ribavirin demonstrated protective efficacy in animal models, their substantially higher inhibitory concentrations suggest lower intrinsic antiviral potency. Importantly, in vitro antiviral potency alone does not necessarily predict therapeutic efficacy in vivo. When in vivo efficacy is considered, the relative performance of these compounds changes substantially. Despite its comparatively modest in vitro activity, favipiravir significantly protected Syrian hamsters by limiting viral dissemination to peripheral organs and the central nervous system. Likewise, ribavirin reduced viral loads and provided partial protection, although it failed to achieve complete viral clearance. Despite these encouraging findings, further preclinical investigation remains necessary before clinical translation can be considered. Although high viral replication has been demonstrated in human placental explants and three-dimensional brain organoids, correlating with manifestations such as vertical transmission and neuroinvasive disease, these experimental systems cannot fully recapitulate the complex circulatory, immunological, and multi-organ interactions that occur during human infection.

This limitation is further compounded by shortcomings in currently available animal models. Immunocompetent adult mice are naturally resistant to severe OROV disease, necessitating the use of immunocompromised strains, such as IFNAR−/− mice, which do not fully reproduce intact human immune responses. Syrian hamsters provide a naturally susceptible alternative but remain limited by the availability of immunological reagents for detailed immune characterization. Non-human primates, including rhesus and pigtail macaques, support efficient viral replication and develop immune responses that closely resemble those observed in humans. However, they generally remain clinically asymptomatic, limiting their utility for studies of disease pathogenesis. Furthermore, their use is constrained by high costs and important ethical considerations, despite their value for evaluating vaccine safety and efficacy.

Several vaccine platforms are currently under development, including mRNA-LNP vaccines, VSV-based chimeric vaccines, and live-attenuated NSs/NSm-deficient viruses. Recent studies have demonstrated that lineage-matched mRNA-LNP vaccines encoding OROV glycoproteins provide complete protection in murine models. Nevertheless, prototype-based vaccines derived from BeAn19991 may provide only partial cross-protection against recently emerged reassorting epidemic strains because of subtle but immunologically relevant antigenic differences within the Gc. These findings underscore the urgent need for flexible vaccine platforms that can be rapidly updated based on real-time genomic surveillance.

Similarly, the contributions of innate and adaptive immune responses, particularly those mediated by natural killer cells and T lymphocytes, to protection against OROV infection remain poorly understood. Future studies should investigate the potential occurrence of antibody-dependent enhancement through in vivo reinfection models and cross-neutralization assays involving phylogenetically related orthobunyaviruses. A more comprehensive understanding of the immune mechanisms underlying OROV infection will be essential for guiding the development of vaccines capable of inducing durable, broad, and cross-protective immunity against genetically diverse OROV lineages.

Taken together, these challenges underscore the urgent need for integrated, multidisciplinary, and sustained research efforts focused on OROV. Progress in this field will depend not only on continued scientific and technological advances but also on interdisciplinary collaboration and international cooperation to mitigate the geographic expansion of OROV. Strengthening surveillance systems, investing in adaptable and scalable technological platforms, and ensuring equitable access to future antiviral therapies and vaccines will be fundamental to reducing the public health burden of this neglected emerging arbovirus.

## Figures and Tables

**Table 1:** Potential direct-acting antivirals against OROV RdRp.

Antiviral Drug/ Class	In Vitro: IC_50_/EC_50_ and CC_50_	In Vivo: Used Dose	Relevant Insights	Study Limitations	References
Favipiravir (T-705)/ Guanosine nucleoside analogue	Huh7 cells: IC_50_ = 90.9 µM No cytotoxicity > 1000 µM Vero cells: EC_50_ = 28.4 µM CC_50_ = 3122 ± 106 µM	Syrian hamster: 600 mg/kg/day	Reducing viral load, preserving cellular morphology, and decreasing CPE in low doses. In vivo, preventing viral dissemination to peripheral organs and the central nervous system	Observed effects were achieved using a recombinant rOROV/GFP reporter systems, which might affect replication efficiency. Treatment fully protected up to 24 hpi	[32,43]
4′-Fluorouridine (4′-FlU)/ Ribonucleoside analogue	Vero cells: EC_50_ = 0.012 µM CC_50_ > 50 µM A549 cells: EC_50_ = 0.019 µM CC_50_ > 50 µM JEG-3 cells: EC_50_= 0.048 µM CC_50_ > 50 µM	AG129 mouse: 10 mg/kg/day	Exhibited potent in vitro antiviral activity and provided complete in vivo protection, reducing viral burden and ensuring 100% survival	Lack of human clinical data, incomplete assessment of long-term resistance development, and the absence of safety evaluations in vulnerable populations, including pregnant individuals and immunocompromised hosts	[44,45]
Ribavirin (RBV)/ Guanosine nucleoside analogue	Huh7 cells: IC_50_ = 10.5 µM No cytotoxicity > 1000 µM Vero cells: EC_50_ = 115.7 µM CC_50_ = 3767 ± 321µM	Syrian hamster: 100 mg/kg/day	Antiviral activity in vitro and provided partial in vivo protection, reducing viral loads in peripheral tissues and reducing pathogenic host responses	Antiviral potency was variable across experimental models, requiring relatively high concentrations and failing to achieve complete viral clearance, particularly from the central nervous system	[32,43,45]
Remdesivir/ Prodrug Adenosine nucleotide analogue	Huh7 cells: IC_50_ = 0.31 µM No cytotoxicity > 10 µM	-	High antiviral potency against multiple OROV strains and superior in vitro efficacy among tested RdRp inhibitors	Confirmation of the proposed antiviral mechanism of action, validation in an in vivo model, and evaluation of long-term efficacy and the development of resistance are required	[32]
Monulpiravir /Cytidine nucleoside analogue	Human liver- derived organoids: IC_50_ = 1 to 2.2 µM CC_50_ > 50 µM	-	Potential cellular model for OROV pathogenesis. Effective antiviral action against multiple OROV strains and potential use of combination therapies	The cellular model does not fully represent the liver due to limitations in cell composition and tissue architecture. Validation of the antiviral effect in an in vivo model and elucidation of the mechanism of action are required	[46]
Acridone (FAC21 and FAC22)/ Synthetic heterocycles	Vero cells: FAC21: EC_50_ = 33.03 ± 4.02 μM CC_50_ = 1640 µM FAC22: EC_50_ = 21.57 ± 3.83 μM CC_50_ = 1528 µM	-	Identification of the viral endonuclease as a promising therapeutic target through combined in vitro and computational analyses, inhibiting viral replication by up to 99.9%	Lack of in vivo validation, pharmacokinetic characterization, and safety assessment in animal models	[47]

**Table 2:** Natural antiviral candidates against OROV.

Natural Antiviral Compound/ Class	In Vitro: IC_50_/EC_50_ and CC_50_	Relevant Insights	Study Limitations	References
Quercetin Hydrate/ Flavonoid	Vero cells: EC_50_ = 53.2 μM ± 26.5 93.6 ± 12.3% viable cells at 500 µM	Anti-OROV activity in vitro with low cytotoxicity and provided mechanistic insights through molecular docking, identifying the viral Gc as a potential antiviral target	Lack of in vivo validation and experimental confirmation of the proposed mechanism of action	[60]
Lysergol/ Alkaloid	Huh7 cells: EC_50_ < 0.156 μM CC_50_ > 40 μM T24 cells: Antiviral activity at 0.6 μM	Potent dose-dependent antiviral activity against different OROV strains, a high barrier to resistance, low cytotoxicity, and a suggested mechanism involving inhibition of the viral RdRp/N protein complex	No in vivo efficacy data or pharmacokinetic and safety characterization, particularly regarding ergot alkaloid-associated vasoconstrictor and uterotonic effects	[61]
Wedelolactone/ Coumestan	Vero cells: IC_50_ = 310 ± 8 nM EC_50_ = 18.92 ± 9.4 µM CC_50_ = 373.5 ± 154.2 µM	Potent inhibition of viral endonuclease activity, dose-dependent suppression of viral replication, and low cytotoxicity in vitro	It requires in vivo validation, pharmacokinetic characterization, and comprehensive safety evaluation	[62]
Acylphloroglucinols and Xanthohumol/ Polyphenolic compounds and Prenylated flavonoid	Vero cells: EC_50_ = 26.7 µg/mL and 96.4 ± 12.1% viable cells at 250 µg/mL (Acylphloroglucinols) and EC_50_ = 50.2 µg/mL and 97.6 ± 15.8% viable cells at 250 µg/mL (Xanthohumol)	Anti-OROV activity in vitro and were supported by molecular dynamics analyses, suggesting interference with multiple stages of the viral replication cycle	Lack of in vivo evaluation, pharmacokinetic characterization, and mechanistic confirmation of the predicted molecular targets	[63]

**Table 3:** Comparison between antiviral synthetic drugs and natural compounds against OROV.

Feature	Synthetic Drugs	Natural Compounds
Potency range	Mainly nanomolar to low micromolar	Mainly low to moderate micromolar
Most antiviral effect compounds	4′-FlU (EC_50_ = 0.012–0.048 µM), Remdesivir (IC_50_ = 0.31 µM)) and Molnupiravir (IC_50_ = 1 to 2.2 µM)	Lysergol (EC_50_ < 0.156 μM) and Wedelolactone (EC_50_ ≈ 19 µM)
Mechanism of action	Direct inhibition of viral replication (RdRp targeting)	Glycoprotein C, RdRp or N Protein targeting
Cytotoxicity	Generally low in vitro	Generally low in vitro
Evidence level	Experimental support	Mostly early-stage studies
Main limitation	Limited OROV-specific clinical data	Lack of pharmacokinetic, toxicity, and in vivo studies

## Data Availability

No new data were created or analyzed in this study. Data sharing is not applicable to this article.

## Abbreviations

The following abbreviations are used in this manuscript:

4′-FlU	4′-fluorouridine
CPE	Cytopathic effect
cRNA	Complementary RNA
dpi	Days post-infection
dsRNA	Double-stranded RNA
FASLG	Fas Ligand
FCCR3A	Fc Gamma Receptor IIIa
Gc	Glycoprotein C
Gn	Glycoprotein N
gRNA	Genomic RNA
HCV	Hepatitis C virus
hpi	Hours post-infection
IF16	Interferon Alpha Inducible Protein 6
IFIT1	Interferon-Induced Protein with Tetratricopeptide Repeats 1
IFN-I	Type I interferon
IFN-α	Interferon-alpha
IFN-β	Interferon-beta
IL-10	Interleukin 10
ISG15	Interferon-Stimulated Gene 15
ISGs	Interferon-stimulated genes
Large	L segment
LLOQ	Lower limit of quantification
Lrp1	Low-density lipoprotein receptor-related protein 1
Medium	M segment
mRNA-LNP	mRNA-lipid nanoparticle
MX1	MX Dynamin-Like GTPase 1
N	Nucleocapsid
NHC	β-D-N4-hydroxycytidine triphosphate
NSm	Non-structural protein of the M Segment
NSs	Non-structural protein of the S Segment
OROV	Oropouche virus
PTPRC	Protein Tyrosine Phosphatase Receptor Type C
RBV	Ribavirin
RdRp	RNA-dependent RNA polymerase
RMP	Remdesivir monophosphate
RTR	Triphosphate form
SARS-CoV-2	Severe acute respiratory syndrome coronavirus 2
SFTS	Severe Fever with Thrombocytopenia Syndrome
Small	S segment
SgRNA	Subgenomic mRNAs
vRNP	Viral ribonucleoprotein complex
VSV	Vesicular stomatitis virus
WDL	Wedelolactone
